# Efficacy and Safety of Acupuncture Combined with Herbal Medicine in Treating Gouty Arthritis: Meta-Analysis of Randomized Controlled Trials

**DOI:** 10.1155/2021/8161731

**Published:** 2021-12-30

**Authors:** Huan Liang, Yan Wu, Wei Zhang, Pin Deng, Fa-Sen Huang, Xin Du, Zhao-jun Chen, Yu-Feng Ma

**Affiliations:** ^1^School of Graduates, Beijing University of Chinese Medicine, Beijing, China; ^2^Beijing University of Chinese Medicine Third Affiliated Hospital, Beijing, China; ^3^Department of Acupuncture and Moxibustion, Capital Medical University Affiliated Beijing Hospital of Traditional Chinese Medicine, Beijing, China

## Abstract

**Background:**

Gouty arthritis is a common metabolic disease caused by long-term purine metabolism and elevated serum uric acid. In recent years, the incidence of gouty arthritis has been increasing year by year. As an effective method for treating gouty arthritis, acupuncture combined with herbal medicine has been widely used in clinical practice. However, the evidence for the treatment needs to be evaluated through systematic review and meta-analysis. Methods. The Cochrane Library, PubMed, Web of Science, EMBASE, China CBM database, Clinical Trials, CNKI, China Wanfang database, and VIP information database were searched from the establishment of each database to March 2021. Randomized controlled trials (RCTs) were included in the study, and the therapeutic effects of acupuncture combined with herbal medicine *versus* conventional therapy, or acupuncture combined with herbal medicine *versus* anti-inflammatory drugs, or acupuncture combined with herbal medicine *versus* acupuncture/herbal medicine alone were compared in the subjects with gouty arthritis. Two authors screened all references, assessed the risk of bias, and independently extracted the data. The binary outcome was summarized using 95% confidence intervals (CIs) and risk ratios (RRs). The overall quality of the evidence was assessed with hierarchy, and meta-analysis was performed with a random-effects model.

**Results:**

A total of 14 randomized controlled trials (1,065 participants, 540 treatment groups, and 525 control groups) with treatment courses of 5 to 21 days were included. Acupuncture combined with herbal medicine and acupuncture was compared in three trials, acupuncture combined with herbal medicine and conventional therapy was compared in 14 of them, and acupuncture combined with herbal medicine and anti-inflammatory drugs was compared in 8 of them. The clinical efficacy (clinical symptoms, serological tests, and visual analogue scale (VAS) results) was significantly improved in the acupuncture combined with herbal medicine treatment group (*P*=0.0005, 95% CI 0.03 to 0.13; 687 participants; 8 trials), and the efficacy in reducing uric acid was also better (*P* < 0.00001; 95% CI −102.89, −68.37; 100 participants; 2 trials; evidence with moderate quality). The effect of acupuncture combined with herbal medicine was better than that of acupuncture alone (RR 1.22, 95%CI 1.06 to 1.41; 139 participants; 3 trials), the effect of acupuncture combined with herbal medicine was better than that of herbal medicine alone (RR 1.31 95%CI 1.08 to 1.57, 100 participants, 2 trials, evidence with moderate quality), and the effect of acupuncture combined with herbal medicine was better than that of colchicine (*P* = 0.02, RR 1.14 95%CI 1.02 to 1.27, 2 trials, evidence with moderate quality). The incidence of adverse events was considerably different between the two groups, and the acupuncture combined with herbal medicine group was significantly superior to the control group in terms of adverse events (*P* < 0.00001; 95% CI (0.08 to 0.32)).

**Conclusions:**

The efficacy of acupuncture combined with herbal medicine was better than conventional drug therapy in treating gouty arthritis. The study results must be interpreted with caution due to the high or unclear risk of bias of the trials included in the study. PROSPERO registration number: CRD42020202544. INPLASY registration number: 202090006.

## 1. Introduction

Gouty arthritis is one of the most common clinical conditions, which account for 5% of all arthritis [1–3]. It is mainly caused by purine disorders and tissue damage induced by elevated serum uric acid [4]. It can have serious adverse effects on the physical and mental health of subjects and their normal life. Therefore, it is necessary to explore the best way to treat gouty arthritis [5]. The clinical trials have shown that the use of nonsteroidal anti-inflammatory drugs (NSAIDs) and corticosteroids can significantly improve clinical symptoms [6–8].

Nevertheless, long-term use of this approach [9] can probably lead to an increase in drug resistance and a decrease in treatment efficacy [10–14]. Although there are no evidence-based guidelines, many patients with gouty arthritis seek acupuncture and herbal remedies, which have been used with safety and efficacy for quite a long time [15–17]. Acupuncture is a treatment method that stimulates specific acupoints on the body surface. It has been used for the prevention and treatment of disease in other eastern countries and China for thousands of years [18].

In ancient traditional Chinese medicine, acupuncture and herbal medicine were believed to work by stimulating acupoints and maintaining a balance between Yin and Yang to mediate the circulation of Qi and blood [19]. In Western medicine, traditional Chinese medicine treatment mechanisms have not yet been well established [20]. Human and animal studies have shown that acupuncture and herbal medicine may play a positive role in reducing blood uric acid, reducing the expression of inflammatory factors, relieving pain, and improving clinical symptoms [21–25].

There has been more and more research on acupuncture and herbal medicine for gouty arthritis in recent years. Several studies reported that acupuncture combined with herbal medicine can promote the dissolution of blood uric acid, increase the clearance of blood uric acid from the kidney [26], and improve treatment efficacy [27–30]. An exploratory systematic review and meta-analysis of acupuncture for the treatment of gouty arthritis published in 2016 found that acupuncture is associated with decreased serum uric acid concentration (*P* < 0.05) [31]. Nevertheless, it is not vital to recommend the methodology because its quality in these reviews is not good enough [32].

Therefore, the purpose of this systematic review was to evaluate the safety and efficacy of acupuncture combined with herbal medicine in the treatment of gouty arthritis and to compare acupuncture combined with herbal medicine and conventional therapy or herbal medicine/acupuncture therapy alone to guide clinical treatment. To ensure the accuracy of these systematic review and meta-analysis, the results of this study needed to be as consistent as possible with the reporting project stated in the Preferred Reporting Items for Systematic Reviews and Meta-Analyses (PRISMA) [33].

### 1.1. Search Methods

The Cochrane Library, PubMed, Web of Science, EMBASE, China CBM database, Clinical Trials, CNKI, China Wanfang database, and VIP information database were searched (as of March 2021). The therapeutic effect of acupuncture combined with herbal medicine was compared (e.g., acupuncture included electroacupuncture, three-edged needle bloodletting, and auricular acupuncture, and herbal medicine included Chinese patent medicine and traditional Chinese medicine). Search terms related to acupuncture, traditional Chinese medicine, gouty arthritis, and randomized controlled trials were set, and the searched literature was limited to clinical studies published in Chinese and English.

### 1.2. Eligibility Criteria

1. Study type: only randomized controlled studies were included.
2. Subject type: the subjects diagnosed with gouty arthritis were included according to clear diagnostic criteria or references, with no restrictions on course of the disease, gender, age, or ethnicity.
3. Type of intervention: the intervention method was composed of acupuncture combined with herbal medicine. The original literature needed to have a specific description of the application process of acupuncture and herbal medicine, such as sterilization, acupuncture manipulation, post-treatment, dosage form, medication method, dosage, and a specific course of treatment.
4. Type of control: the controlled measures should be standard drugs, and the medication method, treatment course, and dosage should be clearly described.
5. Types of observed outcome indicators: clinical efficacies (improvement of clinical symptoms and serological test results) were mainly observed, and adverse effects were concerned with secondary outcome indicators.

In this review, clinical studies of acupuncture combined with Western medicine or needle knife combined with herbal medicine were excluded. Reviews, case reports, retrospective studies, or clinical studies without predetermined results were excluded. For duplicate studies, the author of the study was contacted to resolve any ambiguity. If the author of the study cannot be contacted, the first published study was assumed to be original. If the control group did not implement the regular treatment for gouty arthritis, it was not included in the study. Two reviewers (H Liang and FS Huang) independently selected the randomized controlled trials to be included in the study, and a flow chart to choose the included studies was designed according to the requirements of PRISMA. Much effort was made for this review to find out the ethical approval numbers of the included studies, but unfortunately, no information on ethical approval numbers was available. Considering that this review was a meta-analysis, which pertained to a secondary study, the ethical requirements were not applicable. However, the lack of clear ethical criteria in clinical studies was a factor of bias.

### 1.3. Data Extraction and Management

The literature data were extracted into Microsoft Excel 2013, and the information collected was as follows:

(1) The basic information of the included literature studies: ID (year of publication, initial of the first author, and year), sample size, language, course of treatment, control measures, and intervention measures; (2) basic information of the subjects: gender, age, severity of the disease, course of the disease, and stage of disease; (3) outcome measurement: primary observed outcomes: clinical efficacy and inflammatory serological factors; observed secondary outcomes: adverse effects.

The authors of this review (P Deng and JL Han) independently used the Cochrane-risk-of-bias assessment tool to determine the risk of bias of each included study [34]. If there were any discrepancies, a third review author (H Liang) was consulted to address the problem. The following items were assessed according to “unclear,” “low,” or “high” risk: random sequence generation, blinded outcome assessment, blinded subject and personnel, allocation concealment, selective reporting, incomplete outcome data, and other biases.

### 1.4. Data Synthesis and Analysis

Review Manager Meta 5.3 analysis software was used for data analysis. For clinical efficacy, adverse events, and serological measures, they were shown with the data with 95% confidence intervals (CIs) of risk ratio (RR) (postintervention values used to calculate the efficacy estimates). Statistical analysis was performed according to the latest Cochrane Handbook for Systematic Reviews of Interventions [31]. The meta-analysis would be achieved if the trials were well homogeneous in terms of participants, study design, intervention, control, and outcome. Statistical heterogeneity was calculated using the Higgins *I*^2^ statistic. If there was any significant heterogeneity among studies (*I*^2^>75%), the meta-analysis would not be performed, and the source of the heterogeneity would be assessed. If more than 10 randomized controlled trials tested the same results in a single meta-analysis, a trim-and-fill analysis was used to intuitively assess and publish the bias. The overall quality of the included research evidence was assessed with hierarchy [35]. The subgroup analysis was performed for different types of controls.

## 2. Results

### 2.1. Study Description

**Figure 1:**
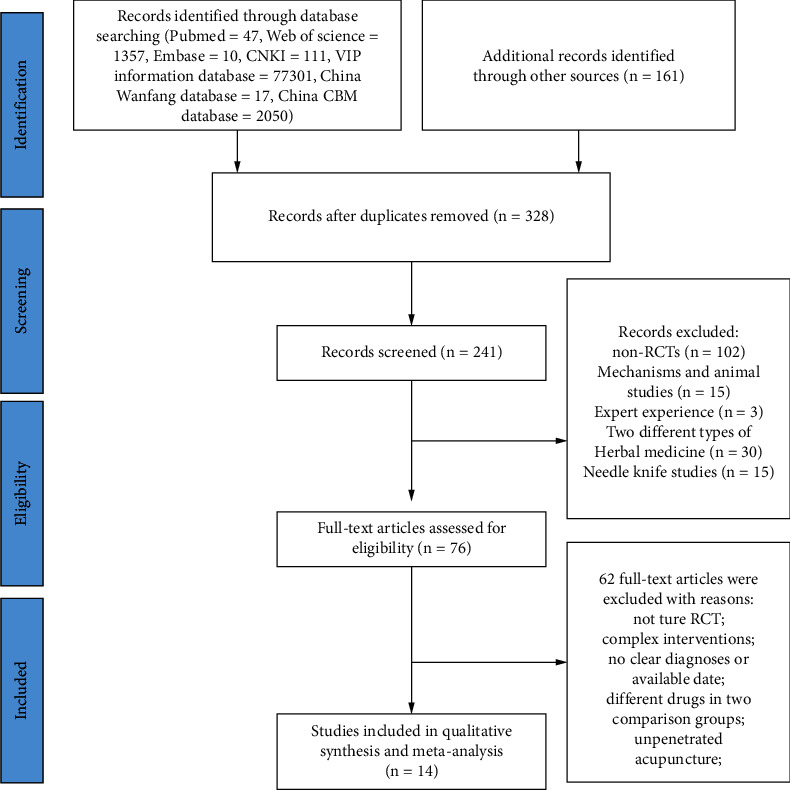
Flow chart for the selection of trials. Flow diagram following the Preferred Reporting Items for Systematic Review and Meta-Analyses (PRISMA) statement.

A total of 569 pieces of literature were searched, and 76 remained after filtering the titles and abstracts. The full texts of the 76 pieces were read, 62 were excluded, and finally, 14 were obtained [36–49]. The filtering process is shown in Figure 1. Basic information of included trials was as follows: 14 randomized controlled trials were included (1,065 participants, 540 treatment groups, and 525 control groups), all the trials were conducted in China, including 13 studies written in Chinese and 1 study reported in English, 8 of them compared acupuncture combined with herbal medicine and anti-inflammatory drugs [39, 40, 41, 43, 44, 45, 47, 49], and 14 compared acupuncture combined with herbal medicine and conventional therapies [36–49]. Three treatment groups were divided into the other 2 trials [48, 49]. The course of treatment was 6–7 days. The participants aged from 18 to 80 years old. The disease course was 3 days to 22 years. There were 729 men and 336 women. The characteristics of the participants included in the trials are shown in Table 1. The details of intervention in acupuncture groups and control groups are shown in Table 2.

### 2.2. Risk of Bias in Included Trials

**Figure 2:**
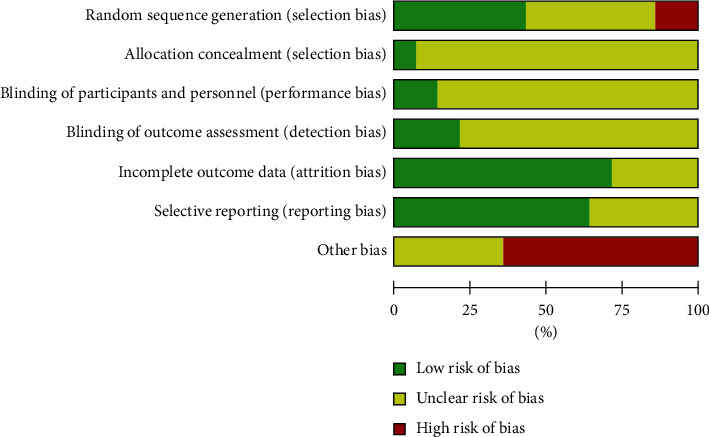
Risk of bias of randomized clinical trials of acupuncture combined with herbal medicine for gouty arthritis. Note: A: Selection bias; B: selection bias C; performance bias of participants and personnel; D: detection bias; E: attrition bias; F: reporting bias; and G: other biases.

A total of six studies reported that the participants were randomized with the random number table and block randomization. These six trials were considered to have a low risk of bias [38, 41, 43, 44, 48, 49]. Only one study used the card approach and sufficient allocation concealment [49]. Therefore, it was supposed to have a low risk of bias. None of them reported whether the assessments of subjects and outcomes were blinded, which were considered as undefined risk of biases. In terms of other biases, 6 showed that if the whole trial was completed by one author, the risk of bias would be greater [36, 38, 42, 44, 47, 49]. Details of the risk assessment deviations of the included studies are shown in Figure 2. In addition, the dot plot was used to describe the risk of bias for each study combined with the forest plot.

### 2.3. Primary Outcomes

#### 2.3.1. Clinical Effect

The criteria for clinical effect referred to the consensus on the diagnosis and treatment of gouty arthritis [51]. Clinical symptoms and serological examination were used as criteria for efficacy evaluation. Clinical outcomes were reported in all included studies.

#### 2.3.2. Acupuncture Combined with Herb versus Conventional Medicine

**Figure 3:**
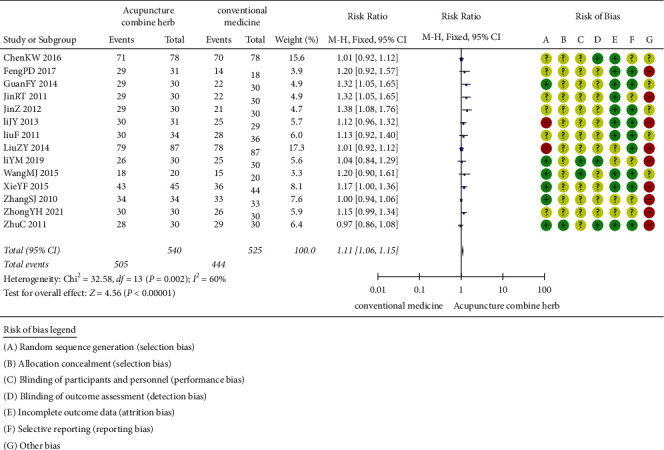
Clinical effect of acupuncture combined with herbal medicine versus conventional medicine.

The treatment efficacy of acupuncture combined with herbal medicine and conventional therapy was compared in 14 trials (involving 1,065 subjects) [36–49]. The duration of the trials ranged from 5 to 21 days. The treatment efficacy of acupuncture combined with herbal medicine was 1.11 times that of conventional therapy (RR 1.11) (Figure 3). There was statistical heterogeneity among studies, and the clinical treatment efficacy of acupuncture combined with the herbal medicine group was better than that of conventional therapy (*P* < 0.00001; 95%CI 1.06, 1.15).

#### 2.3.3. Acupuncture Combined with Herbal Medicine versus Acupuncture

**Figure 4:**
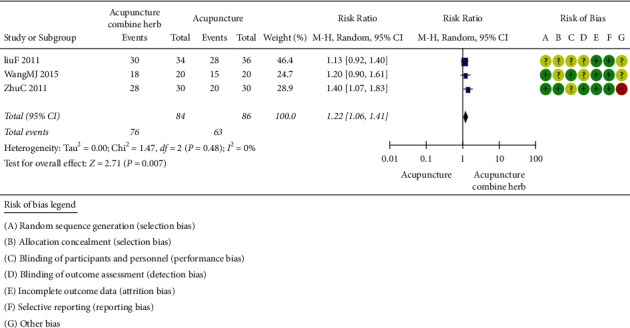
Clinical effect of acupuncture combined with herbal medicine versus acupuncture alone.

The treatment efficacy of acupuncture combined with herbal medicine and acupuncture was compared in three trials (involving 170 subjects) [37, 48, 49]. The number of participants ranged from 60 to 90, and the duration of the trials ranged from 5 to 7 days. As shown in Figure 4, there was no statistical heterogeneity between studies. The clinical treatment efficacy of acupuncture combined with herbal medicine was better than that of acupuncture alone (*P*=0.007, RR 1.22, 95%CI 1.06 to 1.41).

#### 2.3.4. Acupuncture Combined with Herbal Medicine versus Herbal Medicine

**Figure 5:**
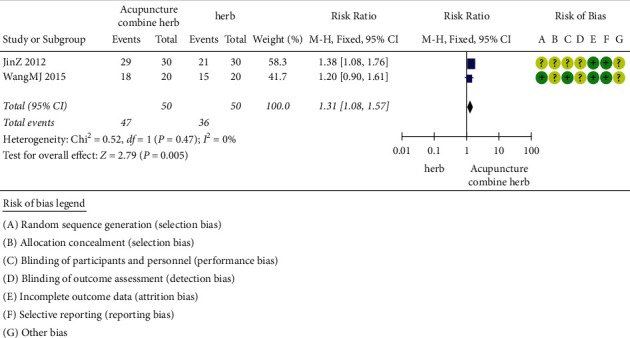
Clinical effect of acupuncture combined with herbal medicine versus herb alone.

The treatment efficacy of acupuncture combined with herbal medicine and herbal medicine alone was compared in two trials (involving 83 subjects) [46, 48]. The number of participants ranged from 60 to 70, and the duration of the trials was 7 days on average. As shown in Figure 5, there was no statistical heterogeneity between studies. The clinical efficacy of acupuncture combined with herbal medicine was 1.31 times that of herbal medicine alone (RR 1.31), and the performance of acupuncture combined with herbal medicine group was better than the herbal medicine alone group in the aspect of the clinical efficacy improvement (*P*=0.005, RR 1.31, 95%CI 1.08 to 1.57).

#### 2.3.5. Acupuncture Combined with Herbal Medicine versus Anti-Inflammatory Medications

**Figure 6:**
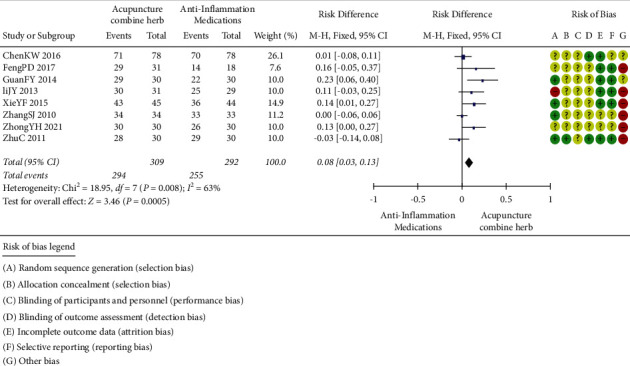
Clinical effect of acupuncture combined with herbal medicine versus anti-inflammatory medications.

The treatment efficacy of acupuncture combined with herbal medicine and anti-inflammatory drugs was compared in eight trials (involving 549 subjects) [39, 40, 41, 43, 44, 45, 47, 49]. The number of participants ranged from 48 to 156, and the duration of the trials ranged from 3 to 21 days. As shown in Figure 6, there was statistical heterogeneity between studies, so the fixed-effects model was used. The clinical treatment efficacy of acupuncture combined with herbal medicine was better than that of anti-inflammatory drugs (*P*=0.0005, 95%CI 0.03 to 0.13).

#### 2.3.6. Acupuncture Combined with Herbal Medicine *versus* Colchicine

**Figure 7:**
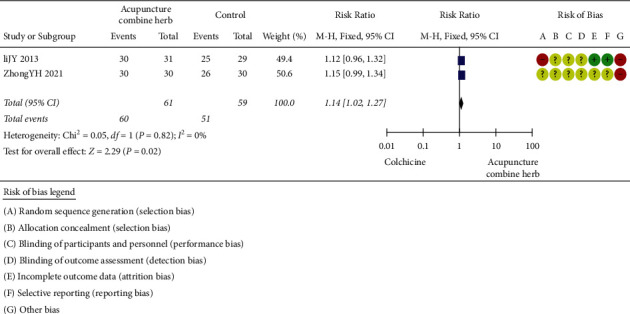
Clinical effect of acupuncture combined with herbal medicine versus colchicine.

The efficacy of acupuncture combined with herbal medicine and colchicine was compared in two trials (111 patients) [39, 47]. The number of participants ranged from 59 to 60, and the duration of the trials ranged from 14 to 21 days. As shown in Figure 7, there was no statistical heterogeneity between studies, so the random-effects model was applied. The clinical treatment efficacy of the acupuncture combined with herbal medicine was better than that of colchicine (*P*=0.02, RR 1.1495%CI 1.02 to 1.27).

#### 2.3.7. Uric Acid (UA)

**Figure 8:**
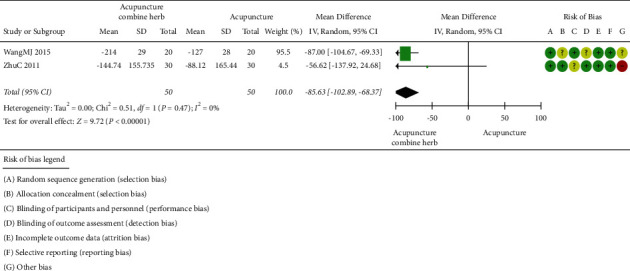
Uric acid of acupuncture combined with herbal medicine versus acupuncture alone.

*(1) Acupuncture combined with herbal medicine versus acupuncture*. The efficacy of acupuncture combined with herbal medicine and acupuncture in reducing uric acid was compared in two trials (involving 100 subjects) [48, 49]. The number of participants ranged from 40 to 60, and the duration of the trials ranged from 6 to 7 days. As shown in Figure 8, there was no statistical heterogeneity between studies, so the random-effects model was applied. The clinical treatment efficacy of acupuncture combined with herbal medicine was better than that of the acupuncture (*P* < 0.00001; 95% CI (102.89, 68.37)).

#### 2.3.8. Visual Analogue Scale (VAS)

**Figure 9:**
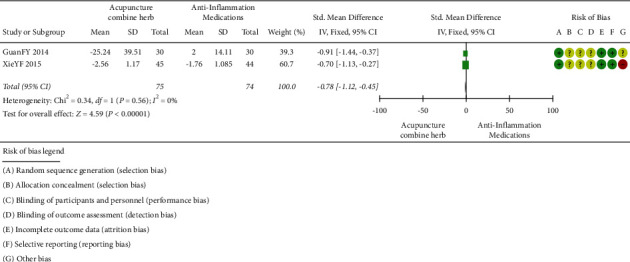
VAS of acupuncture combined with herbal medicine versus anti-inflammatory medications.

*(1) Acupuncture combined with herbal medicine versus anti-inflammatory medications*. VAS (involving 149 subjects) was measured in 2 trials [43, 44]. The number of participants ranged from 60 to 89, and the duration of the trials was 7 days on average. As shown in Figure 9, there was no statistical heterogeneity between the studies. Acupuncture combined with herbal medicine was better than the anti-inflammatory drugs for the reduction in VAS score (*P* < 0.00001, mean difference = −0.78; 95% CI (1.12, 0.45)).

### 2.4. Secondary Outcomes

#### 2.4.1. Adverse Events

**Figure 10:**
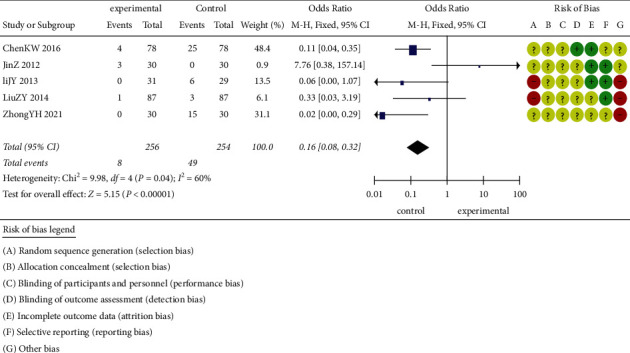
Adverse events of acupuncture combined with herbal medicine compared to conventional medicine.

In the 14 included randomized controlled trials, 5 of them reported adverse events [39, 42, 45–47]. The difference of adverse events between the acupuncture group and the control group is shown in Figure 10. The acupuncture group performed better than the control group in the aspect of the adverse events (*P* < 0.00001; OR = 0.16; 95% CI (0.08 to 0.32)).

#### 2.4.2. Publication Bias

**Figure 11:**
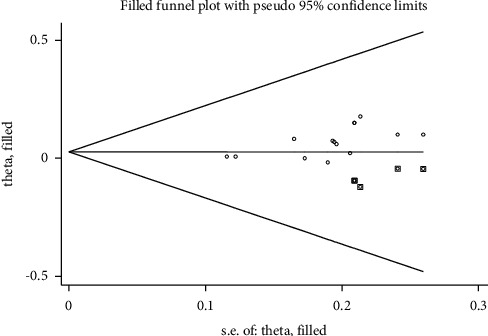
Trim-and-fill analysis for the comparison of clinical treatment efficacy between acupuncture combined with herbal medicine and conventional therapy.

The 14 included studies involved 8 types of comparison and contrast. More than 10 trials compared the treatment efficacy of acupuncture combined with herbal medicine and conventional therapy to treat gouty arthritis, and it could be manifested through trim-and-fill analysis. The publication bias no longer existed when the results were saturated after 5 iterations (*P* > 0.05). Therefore, as shown in Figure 11, this result also indicates that more high-quality studies are needed to verify the conclusions of this review in the future.

## 3. Discussion

Currently, Chinese medicine therapies have attracted increasing attention in the treatment of gouty arthritis, and acupuncture and Chinese herbal medicine are the commonly used therapies in clinical practice. In recent years, there had been a corresponding increase in the study on the treatment of gouty arthritis with acupuncture or herbal therapy [52–54]. Acupuncture is an ancient and effective Chinese medicine treatment method. Modern studies suggest that acupuncture can reduce the release of inflammatory and pain-causing substances, improve the microcirculation in the focal area, and mobilize the immune function of the body, thus stimulating the defense mechanisms of the body [55]. Traditional Chinese medicine treatment plays an essential role in treating gouty arthritis. Some studies included in the review showed that oral or external application of formulas with the main functions of clearing away heat and dampness, promoting blood circulation, and removing blood stasis could effectively relieve the pain of the patients with gouty arthritis and decrease the degree of redness, swelling, heat, and pain of target joints, and traditional Chinese medicine had the advantages of high safety, low incidence of adverse reactions, and individualized treatment [25, 56, 57]. Therefore, acupuncture and traditional Chinese medicine had good clinical application prospects and research value in treating gouty arthritis. There were many studies researched on acupuncture combined with traditional Chinese medicine in treating gouty arthritis, but most of the studies had fewer clinical cases and were single-center studies. Besides, the control groups had several types of treatment, such as acupuncture alone, Chinese medicine alone, and Western medicine alone. No other scholars to date used the method of meta-analysis for system analysis to compare the efficacy of acupuncture combined with Chinese herbal medicine versus Western medicine for gouty arthritis from the perspective of evidence-based medicine. The randomized controlled trials with the intervention group treated by acupuncture combined with herbal medicine were included in this study, the meta-analysis method was used, and 4 treatment options including Chinese herbal medicine alone, acupuncture alone, Western medicine alone, and acupuncture combined with Chinese herbal medicine were involved, which might provide some reference for clinical decision-making in treating gouty arthritis.

A systematic search and a rigorous assessment of the original studies were conducted in this systematic evaluation, with more stringent intervention procedures than other similar studies. This study included 14 RCTs involving 1,065 patients, which aimed to examine the clinical efficacy of acupuncture combined with herbal medicine in treating gouty arthritis. Unlike previous meta-analyses, this study was the first one to include all types of meta-analyses and systematic evaluations on acupuncture combined with herbal medicine for gouty arthritis, which followed the criteria and guidelines of the QUOROM systematic review and meta-analysis [33] with no language restrictions, and multiple literature databases were searched through a comprehensive search strategy. In addition, more trials were added after previous reviews. Thus, this present systematic review differed from previous reviews. This review analyzed clinical efficacy, uric acid, VAS, and adverse effects. Based on the subgroup analysis of the 14 included studies, it was found that acupuncture combined with herbal medicine therapy significantly improved the clinical outcomes of patients compared with acupuncture and herbal medicine therapy alone. Subgroup analysis depicted that acupuncture combined with herbal treatment was superior to conventional treatment, acupuncture alone, herbal therapy alone, anti-inflammatory drugs, and colchicine therapy for improving overall efficiency, relieving pain, and improving signs and symptoms. In terms of improving blood uric acid and VAS scores, acupuncture combined with herbal medicine was much better than acupuncture or anti-inflammatory drug therapy alone. The results of this review were consistent with those of Han et al.'s study in terms of urine acid [58]. These results suggest that acupuncture combined with Chinese herbs can treat gout and reduce the incidence of gouty arthritis. From the point of view of the occurrence of adverse reactions, patients were more likely to have adverse drug reactions in the process of treating gout with Western medicine treatment, which would affect the daily life of patients during the whole treatment process; gouty arthritis patients treated with acupuncture combined with traditional Chinese medicine had fewer adverse reactions, which were mostly caused by the inadaptability of the body to herbal medicine or acupuncture and would gradually disappear during the treatment process. Besides, this review compared acupuncture combined with herbal medicine versus conventional therapy versus Western treatment in a subgroup analysis rather than acupuncture combined with Western medicine therapy versus Western medicine as other meta-analyses did. Therefore, this review could directly compare the efficacy of acupuncture combined with herbal medicine versus Western medicine versus conventional treatment. Blood pricking was the most used acupuncture therapy in the literature included in this study, and a total of 12 studies used this method, accounting for 30% of all acupuncture therapies. The main tools used in blood pricking therapy include three-edged needles and injection needles combined with cupping. The site selection for bloodletting was based on the ashi points and the target joint with recurrent pain, and the volume of blood pricked was about 10–50 ml. This review summarized the clinical effect of acupuncture combined with herbal medicine in treating gouty arthritis and put forward new treatment options.

However, this study has several limitations. First, most included studies presented an unclear risk of bias regarding random sequence generation and allocation concealment. Some of these studies were also at unclear risk of bias in selective reporting and incomplete outcome data. Among the included 14 RCTs, only 6 studies described specific randomization methods, and the remaining studies only mentioned randomization in the articles without a specific description of the randomization method. None of them mentioned allocation concealment and blinding, making it impossible to truly judge their risk of bias. Second, 13 of the 14 were published in Chinese, and 1 was written in English, which reduced the accessibility to other researchers and restricted further research based on these findings. Third, the interventions were not completely consistent among the included studies in this review, such as selected acupuncture points, the formulation and dosage of herbal medicine, and the type of Western medicine. Treating acupuncture combined with herbal treatment and Western medicine treatment as the same intervention method during meta-analysis might cause the bias of the analysis results.

In the subgroup analysis of acupuncture combined with herbal medicine versus conventional treatment, the heterogeneity was higher (I^2^ = 63%) (Figure 3). It is suggested that differences in the type, dose, and duration of therapy of Western drugs in conventional treatment may be responsible for the heterogeneity. After analyzing the publication bias through the trim-and-fill method, it was found that bias no longer existed if the results were saturated after five iterations (*P* > 0.05). This result also indicated that although this study showed that acupuncture combined with herbal medicine was more effective than conventional therapy in treating GA, few studies had high-quality, large-sample, multicenter RCTs. More high-quality studies are needed to verify the findings of this review in the future.

## 4. Conclusions

Based on the evidence in this systematic review, it was found that acupuncture combined with herbal medicine performed better than acupuncture or herbal medicine alone and conventional therapy in terms of improving clinical efficacy, lowering uric acid, and improving VAS score. Due to the generally poor methodological quality of the included trials (based on hierarchical evidence analysis results), the included clinical trials were limited. Hence, the safety and efficacy of acupuncture combined with herbal medicine for the treatment of GA needed to be further validated with high-quality randomized trials. Good trials are the prerequisite for reliable results. To improve the evidence of evidence-based medicine, RCTs should be strictly designed and carried out in the future. Clear and specific randomization methods, allocation concealment, and blinding method should be effectively implemented to control bias. A large sample size is also required. At the same time, in order to improve the reliability of the results to a certain extent, it is recommended to include clinical follow-up to observe the long-term efficacy, report the long-term effectiveness of acupuncture combined with Chinese medicine, and evaluate the quality of life of the patient.

## Figures and Tables

**Table 1:** Characteristics of included randomized trials of acupuncture combined with herbal medicine for gouty arthritis.

Study ID	Year	Gender male/female	Sample size	Age (mean or range, yrs)	Course of disease(mean or range)	Course of treatment, days	Intervention vs. control	Outcomes	Control measures
JinRT 2011	2011	I:29/1 C:28/2	I:30 C:30	I:47.50 ± 10.29 C:43.37 ± 11.34	I:17.62 ± 44.24 C:17.92 ± 35.0	10	Acupuncture plus herbal medicine vs. benzbromarone	Clinical effect, UA, VAS	Benzbromarone: 1 time/day, 50 mg/time
liuF 2011	2011	NR	I:34 C:36	I:40∼60(52) C:40–60(52)	I:2 month-4 years C:2 month-4 years	5	Acupuncture plus herbal medicine vs. acupuncture	Clinical effect	Acupuncture: 1 time/day
liYM 2019	2019	I:21/9 C:23/7	I:30 C:30	I:37∼72 C:35∼70	I:3d-10 years C:7d-12 years	10	Acupuncture plus herbal medicine vs. benzbromarone	Clinical effect	Benzbromarone: 1 time/day, 50 mg/time
ZhongYH 2021	2021	I:28/2 C:29/1	I:30 C:30	I:18–80 (42.33 ± 14.36) C:18–80 (39.30 ± 12.99)	NR	14	Acupuncture plus herbal medicine vs. febuxostat and colchicine	Clinical effect, UA, Scr, BUN, 24 hour urine protein, CRP, TCM score, AEs	Febuxostat: 1 time/day, 40 mg/time; colchicine: 3 times/day, 0.5 mg/time
FengPD 2017	2017	I:22/9 C:12/3	I:31 C:18	I:22–71 C:31–73	NR	14	Acupuncture plus herbal medicine vs. dafen capsules	Clinical effect	Dafen capsules: 1 time/day, 75 mg/time
ZhangSJ 2010	2010	I:34/0 C:33/0	I:34 C:33	32–71 48	7 day-14 years	3–7 days	Acupuncture plus herbal medicine vs. diclofenac sodium enteric-coated tablets	Therapeutic effects	Diclofenac sodium enteric-coated tablets: 3 times/day, 25 mg/time
LiuZY 2014	2014	NR	I:87 C:87	I:44.1 C:43.4	NR	14	Acupuncture plus herbal medicine vs. benzbromarone tablets	Clinicaleffect, CRP, BUA, ESR, VAS, AEs	Benzbromarone: 1 time/day, 50 mg/time
GuanFY 2014	2014	I:28/2 C:29/1	I:30 C:30	I:31–72 (45.3 ± 6.17) C:28–70 (44.7 ± 6.40) C:1–10 (5.76 ± 3.77)years	I:0.5–11 (5.71 ± 3.72) years	7	Acupuncture plus herbal medicine vs. meloxicam and sodium bicarbonate tablets	Clinical effect, UA, VAS,	Meloxicam tablets: 1 time/day, 15 mg/time; sodium bicarbonate tablets: 3 times/day, 1 g/time
XieYF 2015	2015	I:41/4 C:42/2	I:45 C:44	I:40–73 C:42–72	I:1d-14 years C:2d-13 years	7	Fire acupuncture plus herbal medicine vs. diclofenac sodium enteric solution tablet and allopurinol tablet	Clinical effect, VAS,	Diclofenac sodium enteric-coated tablets: 1 time/day, 75–25 mg/time; allopurinol: 1 time/day, 50 mg/time
ChenKW 2016	2016	I:47/31 C:48/30	I:78 C:78	I:(54.1 ± 7.7) C:53.9 ± 8.1	I:(4.1 ± 1.3) years C:(3.9 ± 1.4) years	7	Acupuncture plus herbal medicine vs. diclofenac sodium sustained-release tablets and allopurinol tablets	Clinical effect, adverse triglycerides, cholesterol, high-density lipoprotein, low-density lipoprotein, and AEs	Diclofenac sodium sustained-release tablets: 1 time/day, 75 mg/time; allopurinol: 3 times/day, 100 mg/time
JinZ 2012	2012	I:29/1 C:27/3	I:30 C:30	I:23–67 C:25–66	NR	7	Acupuncture plus herbal medicine vs. gout tablets	Clinical effect, VAS, UA, AES	Gout tablets (Chinese patent medicine): 3 times/day, 0.12 g/time
liJY 2013	2013	I:28/3 C:28/31	I:31 C:29	I:49.67 ± 9.33 C:46.53 ± 10.29	I:4–11(7.1 ± 3.9)d C:2–10(6.7 ± 3.2)d	21	Acupuncture plus herbal medicine vs. colchicine vs. diclofenac sodium sustained-release tablets	Clinical effect, UA, AEs	Colchicine: 1 time/day, 0.5 g/time; diclofenac sodium sustained-release tablets: 1 times/day, 100 mg/time
WangMJ 2015	2015	Male: 35 Female: 25	I:20 C1:20 C2:20	NR	2–13 years	**7**	Acupuncture plus herbal medicine vs. Acupuncture vs. herbal medicine	Clinical effect, UA	Acupuncture: 1 time/day, herbal medicine 2 times/day
ZhuC 2011	2011	I:30 C1:30 C2:30	I:30 C1:30 C2:30	I:48.12 ± 11.21 C1:47.44 ± 11.29 C2:50.36 ± 11.07	I:25.52 ± 9.87 C1:24.16 ± 10.07 C2:26.04 ± 10.03	6	Acupuncture plus herbal medicine vs. indomethacin vs. acupuncture	Clinical effect, UA, ESR,	Indomethacin: 3 times/day, 25 mg/time; herbal medicine: 3 time/day

Note: NR: not reported; I: intervention; C: comparison; Y: yes; N: no; AEs: adverse effects; UA: uric acid; VAS: visual analogue scale; Scr: serum creatinine concentration; BUN: blood urea nitrogen; CRP: C-reactive protein; TCM: traditional Chinese medicine; BUA: blood uric acid; ESR: erythrocyte sedimentation rate; Yrs: years; vs: versus.

**Table 2:** Details of intervention in acupuncture groups and control groups.

Study ID	Acupuncture point [50]	Acupuncture on one or both sides of the body	Duration of each treatment	Frequency	With or without conventional medicine
JinRT 2011	Yinlingquan(SP9), Sanyinjiao(SP6), Zusanli(ST36), Quchi(LI11)	On both sides	30 min	1 time/day;	Y
liuF 2011	Quchi(LI11), Hegu(LI4), Yinbai(SP1), Dadu(SP2), Sanyinjiao(SP6), Yinlingquan(SP9)	NR	30 min	1 time/day;	N
liYM 2019	Taibai(Sp3), Taichong (Liv3), Xingjian(Liv2), Neiting (S44), Xiangu(S43), Qiuxu(G40), intense redness, swelling, and pain areas (usually the location of gouty tophus deposits)	NR	NR	1 time every other day;	Y
ZhongYH 2021	Local joint with recurrent gout	On one side	NR	Acupuncture:1 time;	Y
FengPD 2017	The local tenderness point of the joint lesion and the surface of the distended and bruising vein	On one side	3–5 min	2 times/week	Y
ZhangSJ 2010	Local joint with recurrent gout	NR	2–5 min	3 times/day	Y
LiuZY 2014	Xuehai(SP10), Zusanli(ST36), Sanyinjiao (SP6), Fenglong(S40), Yinlingquan(SP9), Quchi(LI11), Hegu(LI4), Taichong (Liv3), Dadu(SP2), Local ashi point	NR	30 min	1 time/day	Y
GuanFY 2014	Taichong (Liv3), Sanyinjiao(SP6), Zusanli(ST36), Fenglong(S40), Yinlingquan(SP9), Yanglingquan(GB34)	NR	20 min	1 time/day	Y
XieYF 2015	Ashi point	NR	NR	1 time/day	Y
ChenKW 2016	Ashi point(Pain point), Zusanli(ST36), Sanyinjiao(SP6)	NR	15 min	1 time/day	Y
JinZ 2012	Xuanzhong(G39), Sanyinjiao(SP6), Shangqiu(Sp5), Zhaohai(K6), Yanglingquan(GB34), Yinlingquan(SP9), Liangqiu(ST34), Xuehai(SP10), Quchi(LI11), Shaohai (H3), Shousanli(LI10), Chize(L15)	NR	NR	1 time/day	N
liJY 2013	Zusanli(ST36), Sanyinjiao(SP6), Yinlingquan(SP9), Fenglong(S40), Xuehai(SP10), Quchi(LI11), Hegu(LI4), Taichong (Liv3), ashi point	NR	30 min	1 time/day	Y
WangMJ 2015	Zusanli(ST36), Sanyinjiao (SP6), Yinlingquan(SP9), Yanglingquan(GB34), Fenglong(S40), gongsun (SP 4) (both sides), Ashi point	On both sides	30 min	NR	N
ZhuC 2011	Yinbai(SP1), Taichong(Liv3), Sanyinjiao(SP6), Fenglong(S40), Zusanli(ST36), Yinlingquan(SP9), Yanglingquan(GB34), Taibai(Sp3), Hegu(LI4), ashi point	NR	30	1 time every other day;	Y
	Yinlingquan(SP9), Sanyinjiao(SP6), Zusanli(ST36), Quchi(LI11)	On both sides			

Note: NR: not reported; Y: yes; N: no.

## Data Availability

The data used to support the findings of this study are available from the corresponding author upon request.
